# Intravital Assessment of Blood Platelet Function. A Review of the Methodological Approaches with Examples of Studies of Selected Aspects of Blood Platelet Function

**DOI:** 10.3390/ijms21218334

**Published:** 2020-11-06

**Authors:** Dawid Polak, Marcin Talar, Cezary Watala, Tomasz Przygodzki

**Affiliations:** Department of Haemostasis and Haemostatic Disorders, Chair of Biomedical Sciences, Medical University of Lodz, 92-215 Lodz, Poland; dawidpolak1991@gmail.com (D.P.); marcin.talar@umed.lodz.pl (M.T.); cezary.watala@umed.lodz.pl (C.W.)

**Keywords:** platelets, intravital studies, thrombosis, atherosclerosis, inflammation, metastasis

## Abstract

Platelet biology owes to intravital studies not only a better understanding of platelets’ role in primary hemostasis but also findings that platelets are important factors in inflammation and atherosclerosis. Researchers who enter the field of intravital platelet studies may be confused by the heterogeneity of experimental protocols utilized. On the one hand, there are a variety of stimuli used to activate platelet response, and on the other hand there are several approaches to measure the outcome of the activation. A number of possible combinations of activation factors with measurement approaches result in the aforementioned heterogeneity. The aim of this review is to present the most often used protocols in a systematic way depending on the stimulus used to activate platelets. By providing examples of studies performed with each of the protocols, we attempt to explain why a particular combination of stimuli and measurement method was applied to study a given aspect of platelet biology.

## 1. Introduction

Platelets are non-nucleated components of blood. Their best recognized physiological function is primary hemostasis, i.e., formation of the hemostatic plug in the site of blood vessel injury. The plug not only limits blood extravasation, but it also creates a procoagulant surface on which the coagulation cascade develops to form fibrin mesh which stabilizes the plug. Effective formation of the hemostatic plug is assured by the ability of platelet to adhere to the site of injury and by firm binding to adjacent platelets. Such a profound phenotype switch from a circulating blood component to an element of a structure which withholds blood outflow demands significant alterations in platelet biochemistry. These alterations are generally termed platelet activation. This process involves the change of platelet shape, driven by reorganization of cytoskeleton; modification of membrane receptors’ affinity; and release of certain factors, which by acting on adjacent platelets facilitate their activation.

Much of our current understanding of platelet biology and platelet’s contributions to thrombus formation is derived from in vitro or ex vivo experiments in which platelet aggregation in response to selected stimuli or platelet adhesion to various molecules under static or under flow conditions is measured. These approaches provided information on platelet biochemistry and intracellular signaling pathways which are activated upon platelet stimulation. In vitro approaches, however, have certain drawbacks. Platelets are very susceptible to manipulations which accompany the collection of blood samples and their preparation for experimental procedures. Such manipulations, which include blood withdrawal from the vessel and cell isolation either by centrifugation or by gel filtration, may lead to artifactual platelet activation [1]. Moreover, under in vitro conditions, platelets are devoid of the influence of natural suppressors of platelet activation produced by the vascular endothelium, such as nitric oxide and prostacyclin. This may also lead to a bias of the results obtained under in vitro conditions. For these reasons, the knowledge acquired under in vitro conditions is often verified by in vivo studies in animal models.

Platelet biology owes to intravital studies not only a better understanding of platelets’ role in primary hemostasis but also the findings that platelets are important factors in inflammation and atherosclerosis.

There is a heterogeneity among research protocols used in intravital studies of platelets, due to the variety of aspects of platelet function that are studied. The choice of the research protocol is driven first by a question of which aspect of platelet biological activity will be examined. This determines whether platelets should be activated by the researcher prior to measuring their response or whether the researcher is interested in a native state of platelets. If activation is required, a proper stimulus must be chosen to relevantly simulate physiological or pathophysiological conditions to be investigated. Finally, platelet response must be quantified. The method of quantification depends on the manifestation of platelet activity, such as aggregation or adhesion, which is relevant in a given experimental setup.

**Figure 1:**
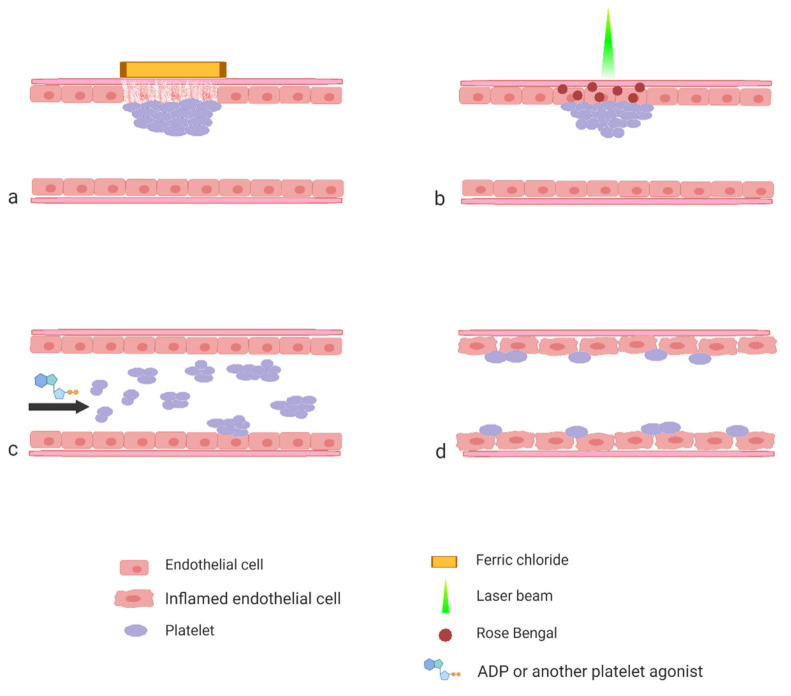
Schematic representation of the experimental models described in the paper. Induction of experimental thrombosis by topical application of FeCl_3_ (**a**) or by laser (**b**); induction of experimental thromboembolism by systemic application of platelet agonist (**c**) and interaction of platelets with inflamed endothelial cells (**d**).

The aim of this review is to present the most often used protocols by showing how they were applied to studies of various aspects of platelet biology. The majority of the protocols that are described in the literature can be roughly divided into three categories based on the procedure applied by the researcher to activate platelet response (Figure 1).

The first category consists of the protocols wherein thrombosis is induced by means of a controlled injury to the vascular wall. The second category consists of protocols wherein platelet activation is triggered by the systemic injection of a specific agonist. Both of the two aforementioned groups of protocols serve to study the mechanisms of thrombosis and to test the efficacy of potential antiplatelet and antithrombotic compounds. The third category, which is the most heterogeneous, includes studies aimed at interactions of platelets with the mechanically intact vascular wall. These protocols are most often applied in studies of the platelet’s role in inflammation, atherosclerosis development or metastasis.

## 2. Thrombosis Triggered by Vessel Injury

Arterial thrombosis is an effect of disruption of the endothelial layer as a consequence of rupture of the atherosclerotic plaque. This process in experimental conditions is mimicked by denudation of the endothelial layer. This can be achieved with mechanical, physical or chemical intervention and may involve all layers of the vessel wall or may be limited solely to the endothelial layer [2]. Formation of thrombus can be quantified either by means of the measurement of blood flow decrease in the injured vessel or can be based on the measurement of thrombus size visualized with the use of intravital microscopy. In the first approach, the measurement of blood flow can be performed with the use of laser or ultrasound Doppler technique. The measurement is based on the Doppler effect occurring mainly upon moving red blood cells inside the vessel. The signal is proportional to the linear velocity of blood stream and decreases when blood flow is slowed down due to occlusive thrombus formation. In the second approach, a thrombus size is estimated based on two or three-dimensional images.

Chemical denudation of the endothelial layer is usually achieved by topical dripping or applying paper soaked with a ferric chloride (FeCl_3_) solution on a surgically exposed blood vessel. The most often used blood vessels include the carotid artery, the femoral artery, the inferior vena cava and the mesenteric or the cremaster vessels. Ferric chloride passes through the endothelium via an endocytic–exocytic pathway into a lumen of a vessel to cause endothelial denudation with clot formation consisting of activated platelets, fibrin and erythrocytes [3].

This approach has been used to study one of the most important aspects of platelet biology in the context of thrombosis, i.e., a contributions of specific platelet receptors to recruitment of platelets to the site of a vessel injury and to the thrombus. Platelet interactions with the vascular wall were examined in the arterial thrombus formation model in vivo in mice under conditions of either a blockade or deficiency of glycoprotein VI (GPVI) [4]. The authors used three models of thrombus formation in the carotid artery: mechanical injury by means of ligation, mechanical injury by the introduction of a wire into the artery and chemical injury with the use of 10% FeCl_3_. Functional blockade of GPVI was achieved by the preincubation of platelets with Fab fragments of anti-GPVI JAQ1 antibodies prior to the infusion of platelets to a recipient mouse. The GPVI-deficiency, in turn, was induced by the administration of JAQ1 five days before the experiment, which led to a proteolytic degradation of GPVI on the platelet surface. Platelets infused to a recipient mouse were at the same time fluorescently labeled to be visualized by fluorescence intravital microscopy. Platelets interacting with the vascular wall were classified according to their patterns of their interaction as tethered platelets or as firmly adherent platelets. The results demonstrated that the blockade or absence of GPVI impairs both initial platelet tethering and stable aggregation at sites of injury. Additionally, the time to thrombus formation was significantly prolonged in both models of GPVI impairment. The above studies proved a major role of GPVI in thrombus development. Since collagen was proven to be a major ligand for this receptor in previous in vitro studies, the authors also concluded that subendothelial collagen is the main trigger for thrombus formation when the endothelial layer is disrupted. Nowadays the knowledge regarding the thrombus build-up and its structure has expanded thanks do the development of microscopic techniques. These new findings are discussed further in the article.

The role of GPIb/V/IX in thrombus formation has been suggested by in vitro experiments [5] and later confirmed in intravital studies. Experiments were performed in transgenic mice, in which the extracellular domain of GPIbα was replaced with another protein, and in mice in which the 45-kDa N-terminal domain of GPIbα was deleted [6]. In mesenteric arterioles of these mice, thrombus formation was induced with the use of 10% FeCl_3_ and monitored by means of intravital microscopy. In both types of transgenic mice, platelets were unable to form a thrombus and platelets from these mice were unable to incorporate into the growing thrombi in wild-type (wt) mice.

The role of this receptor has been further confirmed in vivo in another genetic model of mice with the deletion of an intracellular part of GPIbβ and in mice with the deletion of entire GPIbβ [7]. Thrombus formation was initiated with the use of 20% FeCl_3_ in the carotid artery and the vessel occlusion was monitored with the use of a Doppler probe. The time to occlusion in both strains of mice was extended in comparison to wt mice.

The FeCl_3_-induced thrombosis model has also been used to verify the role of platelet-derived von Willebrand factor (vWF) in thrombosis [8]. For this purpose, the authors used two types of chimeric mice, which expressed vWF only in platelets or which specifically lacked platelet vWF. In these mice and in wt and vWF deficient mice, a thrombus was induced in the carotid artery with the use of 12% FeCl_3_. Blood flow in the artery was monitored with the use of a laser Doppler flowmeter. It was shown that a lack of platelet vWF did not impair the formation of occlusive thrombus in the artery, and that the platelet vWF in the absence of the endothelial vWF was not able to support thrombus formation. These experiments proved that the platelet-derived vWF did not play a role in the course of thrombus formation. Surprisingly, however, in a model of focal cerebral ischemia, mice that expressed vWF only in platelets developed brain infarcts to the same extent as wt mice, which pointed to the undeniably important role of the platelet-derived vWF in inflammatory processes following transient ischemia.

Intravital studies with the use of the FeCl_3_-induced thrombosis model revealed that the platelet activation during the formation of the thrombus is also regulated by cathepsin G secreted by activated neutrophils [9]. This was evidenced by the impaired formation of occlusive thrombus in mesenteric arterioles of mice injected with cathepsin G inhibitor and in cathepsin G-deficient mice. Thrombus was induced with the use of 15% FeCl_3_ in mice infused with platelets labeled with calcein-AM. The formation of thrombus was monitored by means of intravital microscopy. The authors suggest that cathepsin G activates platelets via the cleavage of the protease-activated receptor 4 (PAR4).

Besides being used to understand the mechanisms of thrombus formation, the FeCl_3_ model of thrombosis is also extensively applied to study antithrombotic compounds.

P2Y_12_ inhibitors are a family of antiplatelet drugs which block ADP-dependent platelet activation. It has been shown that P2Y_12_ inhibitors not only limit thrombus formation, but also suppress the formation of neointima. The latter finding is of special importance when one takes into account that neointima formation is a common complication which follows percutaneous coronary interventions [10]. To test the effect of ticagrelor on neointima formation, an injury to the carotid artery in mice was induced with 10% FeCl_3_ in aseptic conditions and was followed for 24 h [11]. Administration of ticagrelor prior and after the injury led to a significant decrease in the size of neointima. A similar study was performed with a single oral dose of prasugrel. Its antithrombotic effect was correlated with a lowered formation of neointimal hyperplasia 21 days after the injury [12]. These studies, besides providing clinically relevant information regarding beneficial effects of P2Y_12_ inhibitors, confirmed that the role of platelets extends beyond hemostasis.

The FeCl_3_ model has been used to test novel pharmacological solutions in antiplatelet therapy. One such potential target is guanylyl cyclase (GC). The enzyme which in physiological conditions is activated by nitric oxide, synthesizes cyclic guanosine monophosphate (cGMP) which diminishes platelet reactivity. Low levels of cinaciguat—guanylyl cyclase activator were found to amplify the antiplatelet effect of prasugrel on thrombosis induced in a mouse’s carotid artery with 10% FeCl_3_ and monitored with the use of ultrasonic Doppler flow probe [13].

BF066 (2-methylthio-6-phenethylaminoadenosine), an adenine derivative, is a compound of a double activity of an adenosine receptor (A_2A_) agonist and a phosphodiesterase inhibitor. It was shown to inhibit thrombus formation induced with the use of 10% FeCl_3_ in the mesenteric arterioles of mice [14]. The growth of thrombus was monitored with the use of intravital microscopy. Interestingly, to quantify the effects of the tested compound, the authors used three parameters characterizing formation of the thrombus: the time to formation of the first thrombus larger than 20 µm; the number of emboli exceeding the size of 20 µm; and finally, the exact sizes of emboli. This approach of quantification is worth noting, as it provides more information on the thrombus formation than the most frequently used methods based solely on measuring the thrombus area. Embolizing thrombi of a given size can occlude distant vessels, thereby showing that a tested compound which decreases the number of such thrombi provides additional information in favor of this compound. Comparing these parameters, the authors concluded that BF066 presented antithrombotic activity equal to clopidogrel given in the same dose.

Another antiplatelet factor, which was tested in the model of FeCl_3_-induced thrombosis, was the modified human soluble calcium-activated nucleotidase (human SCAN). This enzyme is a homologue of the insect salivary anticoagulant apyrases, which are injected by insects feeding on blood to prevent blood clot formation. The insect SCAN acts by decomposing ADP which is a strong platelet activator. Otherwise, human native SCAN lacks the hydrolyzing activity towards ADP. With the use of site-directed mutagenesis, the authors constructed and expressed in bacteria the mutant human SCAN, which possessed the activity of ADP hydrolase and was able to inhibit platelet aggregation in vitro induced by ADP. To prove antithrombotic activity of the protein, the in vivo thrombosis was induced with 10% FeCl_3_ in the jugular vein and blood flow was measured by a laser Doppler probe. Mice injected with the recombinant enzyme were protected from the thrombus formation, which confirmed that the protein has significant antithrombotic activity [15].

As was exemplified above, FeCl_3_ is one of the most often utilized stimuli to induce thrombus formation. This model was used extensively to explain basic mechanisms of the role of platelet receptors during thrombus formation. Despite this, in recent years some concerns have been raised against this method. One of them is a variability between various experimental approaches in the concentration of FeCl_3_ used to trigger thrombus formation. The working concentrations of FeCl_3_ in the literature vary between 10% and 70%. Additionally, a time span of the application varies. These parameters significantly influence the stability of the thrombus. Wang et al. [16] showed how the conditions of thrombus formation affect the susceptibility of thrombi to heparin and clopidogrel in mice. It was found that the doses of these drugs, which were capable of completely inhibiting hemostatic function in the tail bleeding test, were not effective at decreasing the formation of thrombus induced by 5% FeCl_3_. In turn, thrombi formed in response to 2.5% FeCl_3_ were susceptible to heparin and clopidogrel used in doses which were effective in prolonging tail bleeding time. The authors concluded that a lower FeCl_3_ concentration (2.5%) favored the conditions more relevant to test the efficacy of antiplatelet and antithrombotic drugs. Similar experiments were performed in rats [17]. Different concentrations of ferric chloride (5, 10, 20, 40, 60 and 80%) were tested on the rat carotid artery. Blood flow was measured by a pulsed Doppler probe and time till occlusion (TTO) was recorded. The solution of 20% FeCl_3_ was shown as a threshold concentration, which caused the measurable formation of thrombus that was susceptible to antiplatelet drugs, such as aspirin, ticlopidine and clopidogrel, and to anticoagulant drugs, such as heparin and warfarin. Therefore, these authors concluded that 20% FeCl_3_ was an optimal concentration for the examination of the efficacy of anticoagulants and antiplatelet agents in rats.

Serious controversies exist regarding the mechanisms responsible for thrombus generation in the FeCl_3_ model. Dubois et al. [18] studied the role of the collagen receptor GPVI in the arteriolar thrombus formation, which was induced with a severe or mild FeCl_3_ injury. GPVI-depleted mice were used in this study. The authors proved significant differences in the extent of collagen deposition depending on the FeCl_3_ concentration: after a mild (8%) FeCl_3_ injury, the appearance of collagen was lower compared to a severe (10% FeCl_3_) injury. Importantly, however, in both conditions, the rate of thrombus formation was decreased in the GPVI-depleted mice, which confirmed the collagen contribution to platelet recruitment in the FeCl_3_ model.

In contrast, Eckly et al. [3] questioned the role of collagen in this model. In studies performed by these authors, FeCl_3_ was applied at various concentrations (7.5% and 20%) and for different time periods with the use of mouse carotid artery or mesenteric arteries. Thrombus formation was monitored by intravital fluorescence microscopy accompanied by ultrasound Doppler flowmetry to evaluate the time to occlusion. Vessel walls subjected to FeCl_3_ were additionally examined with the use of electron microscopy to visualize the ultrastructures of the injuries and to identify elements of the coagulation cascade present at the site of injury. It was revealed that after the topical application of ferric chloride, ion-rich spherical bodies were formed on the intraluminal surface of the blood vessel wall. The spheres were covered with a tissue factor (TF) and with fibrin fibers before the platelets’ appearance. What is more, no exposition of adhesive proteins present underneath the internal elastic lamina, such as type I collagen, was detected. In line with this, the GPVI depletion of platelets had no effect on thrombus formation in either of the tested FeCl_3_ concentrations. This suggested that thrombin, and not collagen, is a primary activator of thrombus formation in the FeCl_3_-injury model. These findings are in contradiction to the results of Massberg [4] and those of Dubois [18] described above.

A possible explanation for these discrepancies was provided by Ciciliano et al. [19], who suggested a multistep model of thrombus formation in response to FeCl_3_. They distinguished two phases of the clot formation induced by FeCl_3_. In the first phase, red blood cells aggregate when their negatively charged molecules bind to positively charged Fe^3+^ ions. In the next phase, adherent blood components initiate the biological cascade and the formation of a final clot containing red blood cells, platelets and proteins. These results indicate that FeCl_3_-induced thrombosis relies on the complex, multifaceted mechanisms.

Since the induction of thrombus formation with FeCl_3_ raises various controversies, other methods of the thrombus induction are gaining more attraction. One such methods is the use of rose Bengal. Rose Bengal is a popular mildly toxic bright red stain. The ability of this dye to produce reactive oxygen species has been effectively used as an alternative approach to the intravital studies on platelets. The technique mimics the endogenous endothelial injury, the process leading to the development of thrombosis. Rose Bengal is a photosensitizing dye effective for in vivo studies because of its low systemic toxicity and high photochemical efficiency. Its solution is topically either applied onto vessels or injected directly into the bloodstream, where it remains completely inert until it is induced by the monochromatic beam of laser light (wavelength 540–570 nm). Photons generate singlet oxygen and facilitate superoxide anion formation at the site of focal targeting, leading to endothelial cell damage [20]. The advantage of this method is that the injury of the endothelium occurs only at the site at which the laser beam is directed [21].

This technique was used to show the interaction of GPIbα with vWF, the phenomenon which is important not only during the initial platelet tethering to the subendothelial matrix but also during the platelet recruitment and adhesion to already adhered platelets [22]. Thrombi were induced in mesenteric vessels of rats, and when an initial layer of platelets adhering to the site of the injury became formed, a pool of fluorescently labeled platelets preincubated with functional blocking antibodies directed against either GPIbα or α_IIb_β_3_ or GPV was injected. Adhesion of these modified platelets to a pre-formed thrombus was assessed with the use of intravital microscopy. It was found that the functional blockade of GPIbα substantially decreased platelet adhesion toward building a thrombus, pointing at a crucial role of the GPIbα interaction with vWF at this step of thrombus formation.

Laser-induced thrombosis was also used to examine the antiplatelet and anticoagulation effect of sulfated β-O4 lignin (SbO4L) [23]. This compound binds to thrombin exosite 2, which is a binding site for GPIbα. Binding of thrombin to this receptor allows the thrombin to activate protease-activated receptor-1 (PAR-1) on platelets. Therefore, the binding of SbO4L to thrombin blocks the enzyme’s ability to activate platelets via the PAR-1 pathway. What is more, the binding of SbO4L to thrombin also allosterically decreases its proteolytic activity towards fibrinogen. The antithrombotic activity of this compound was tested in the rose Bengal photostimulation model in parallel with the FeCl_3_ model. The measurement of occlusive thrombus formation was monitored with the use of ultrasound Doppler. It was shown in both models that SbO4L significantly decreases formation of occlusive thrombus.

Laser-induced thrombus formation may be also performed without the application of a photosensitizing dye. This is possible when a laser is incorporated in microscope optics and the beam of light used in this method is coherent but not focused. In this way, it can freely pass through the intermediate tissue without damaging it. Only at the site of the focal point of the objective, where a laser beam is focused, does the injury occur. Additionally, the size of injury is limited to the diameter of a laser beam and in this way it is accurately controlled [24].

This approach has been used to test the activity of antiplatelet compounds. With its use it has been shown that acetylsalicylic acid (ASA) supports the antiplatelet activity of clopidogrel but only in doses which do not impair PGI_2_ synthesis [25]. Laser induced formation of thrombi in cremaster arterioles was more effectively inhibited by clopidogrel in combination with 0.15 mg/kg b.w. of ASA than in combination with 0.6 mg/kg b.w. of ASA. At the same time, 0.6 mg/kg b.w. of ASA significantly decreased systemic PGI_2_ synthesis, whereas 0.15 mg/kg b.w. of ASA had no effect on PGI_2._ Based on these finding, the authors emphasized the importance of optimization of ASA dose in dual antiplatelet therapy.

Studies in animal models may be hindered by a divergence between the models and human physiology. Antiplatelet drugs from the group of αIIbβ3 antagonists were shown to be less effective in inhibiting mouse platelets aggregation than that of human platelets [26]. This limits the use of murine models to study the effects of these drugs. A possible way to circumvent this limitation could be an infusion of human platelets to mice. However, interaction of human GPIb with mouse vWF is limited due to the interspecies differences in A1 domain of vWF [27]. Therefore, thrombus formation in such model could be compromised. To manage this problem a genetically modified model of mouse was created which expresses vWF capable to interact with human platelets [26]. Relevance of these mice as a model to test the efficacy of antithrombotic drugs was tested with the use of laser-induced thrombosis in cremaster arterioles. Prior to infusion of human platelets, thrombocytopenia was induced in the experimental mice with the use of specific antibodies. In this model antiplatelet drugs from the group of αIIbβ3 inhibitors, including abciximab, eptifibatide and tirofiban, have shown inhibitory activity in doses similar to these used in humans in contrast to wild-type mice which were non-responsive to such low doses. Therefore, this model could be used to test drugs which, due to interspecies differences in receptor structure, are less effective in murine platelets than in humans. The model has been utilized to test a novel αIIbβ3 antagonist RUC-4 in laser-induced thrombosis in cremaster arterioles [28].

Interesting examples of the application of laser-induced thrombosis were studies aimed at understanding of the time course of the thrombus buildup and its architecture in the mouse [29]. Thrombus was induced in the cremaster arteriole by a pulsed nitrogen dye laser applied through the microscope objective. Mice were injected with platelet-specific anti-CD41 antibodies and anti-fibrin and anti-tissue factor fluorescent-labeled antibodies. High-speed intravital four-channel imaging allowed them to monitor the assembly of the developing thrombus inside the arteriole. Several phases of thrombus growth were differentiated. In the first phase (after 4 s), the accumulation of tissue factor preceded fibrin formation within the thrombus. After 26 s, fibrin appeared and the thrombus increased its volume. Between 34 and 60 s, fibrin was extended through much of the thrombus and downstream end of the thrombus remained entirely built of platelets. These studies provided an important insight into the sequence of events during thrombus formation.

This aspect was studied in more detail by Stalker et al. [30] with the use of a similar laser injury model. The use of a spinning disc confocal microscope and injecting specific antibodies and other fluorescent probes into mice allowed them to assess the three-dimensional structure of the thrombus. Application of fluoro-labeled anti P-selectin antibodies allowed them to show which fraction of thrombus was rich in activated, degranulating platelets. Fluorescently-labeled fibrinogen allowed them to reveal the formation of fibrin. Finally, fluorescently labeled dextrans allowed them to evaluate thrombus porosity. This was achieved by using dextrans of different molecular weight and hence having various abilities to penetrate into the thrombus body. The studies revealed that a hemostatic plug developed a characteristic architecture, in which an inner core of closely packed platelets with high expression of P-selectin was overlaid by an outer shell of less activated, loosely packed platelets. Fibrin was present in the innermost section of the core and in the vessel wall in the proximity of the injury. It was absent in the outer shell region, and in the outer layers of the core region. The use of a thrombin inhibitor hirudin and P2Y_12_ antagonist clopidogrel allowed them to show that ADP and thrombin play differential roles in activating platelets in these two regions. Thrombin plays more important role in the core region, while ADP is mostly responsible for activation of platelets in the outer shell region. Since ADP is a relatively small molecule, it can easily diffuse from shell region of loosely assembled platelets, which inevitably limits thrombus growth. This study showed that thrombus should be considered as a spatially organized structure rather than a mass of fully activated platelets evenly interspersed with fibrin. Such hierarchical organization is responsible, on the one hand, for the stabilization of hemostatic plug, and on the other hand, it provides mechanisms which limit its growth.

In recent years there has been growing interest in the role of protein disulfide isomerases in platelet activation. Laser induced-thrombosis was used to prove a contribution of thiol isomerase ERp5 to thrombus formation [31]. Fluorescently labeled anti-ERp5 or preimmune IgG and anti-CD42b were infused into recipient mice to detect ERp5 in the laser-induced thrombi generated in cremaster arterioles. Isomerase ERp5 was detected at the thrombus site. To evaluate the contribution of ERp5 to thrombus formation, the anti-ERp5 antibody or IgG control was infused into mice, followed by the labeled anti-CD42b and the labeled anti-fibrin-specific antibodies. The integrated fluorescence intensity was calculated for each thrombus generated before and after the infusion of either the anti-ERp5 or control antibody. Thrombi after the anti-ERp5 infusion indicated decreasing deposition of platelets and fibrin compared to the infusion of preimmune IgG. Thus, it was shown that isomerase ERp5 is essential in clot formation upon vascular injury and the authors suggest that it may play its role via association with α_IIb_β_3_ integrin.

In turn, significant differences between the FeCl_3_-induced and laser-induced thrombi in the mechanism of generation were revealed by Dubois et al. [18]. In the laser-induced thrombus model, the formation of thrombi in FcRγ-null mice and in GPVI-depleted mice did not differ when compared to wt mice, which suggested that the collagen-dependent pathway of platelet activation does not occur in this model. In line with this, collagen exposure was undetectable at the sites of laser-induced vascular injury. At the same time, the tissue factor accumulation was higher in thrombi generated in response to laser injury, when compared to that induced by FeCl_3_. It was concluded that in the laser-injury model, the platelet activation by thrombin prevails over that mediated by collagen-GPVI interactions. The authors concluded that the laser-induced injury model is not relevant to study GPVI-dependent anti-thrombotics.

The approaches used to induce vascular injury in vivo, as with all experimental techniques, suffer from certain inadequacies and are subject to controversies and criticism. In the case of the described models of thrombosis, the point of greatest concern is the resemblance of the alterations caused by experimental injuries to these observed in fissuring or rupture of the atherosclerotic plaque. Another problematic aspect is the high variability of the conditions applied to induce injury. These issues have been systematically reviewed and discussed in the excellent review by Jagadeeswaran [32]. There have been recently published review papers focused on the models of ferric chloride–induced thrombosis [33] and on laser-induced thrombosis [34]. The reader is strongly encouraged to refer to these papers to familiarize themselves with details regarding the technical aspects and limitations of these models.

## 3. Platelet Activation by Systemic Application of an Agonist

Injury of the vascular wall leads to the activation of platelets in several inseparable pathways. However, in some studies researchers are interested in assessing the platelet response in vivo to an isolated, defined chemical stimulus rather than to a combination of factors. In such a case, selected agonists are injected into an animal and the platelet response is measured intravitally. This approach to some extent resembles measurements performed in whole blood in vitro, where an isolated stimulus is added to a blood sample and the aggregation of platelets is monitored. An obvious advantage of the in vivo approach is an ability to evaluate whether the platelet aggregates, which are formed as an effect of the used stimulus, are capable of occluding blood vessels in physiological conditions. The use of animals with certain vascular pathology also allows one to test whether pathological conditions affect platelet occlusive properties.

The simplest way of quantification is based on a measurement of a death rate of the animals injected with a tested substance. Since death of animals is presumed to be predominantly an effect of occlusion of pulmonary circulation with platelet aggregates, the death rate was considered proportional to the pro-aggregatory effect of the used agonist. Liang et al. [35] utilized this approach to evaluate antithrombotic properties of pentamethylquercetin (PMQ). Platelets were activated by the tail vein infusion of collagen and epinephrine. Injection of the mixture of collagen and epinephrine resulted in a death rate of 100% the examined mice. As the authors expected, PMQ had an antithrombotic effect and significantly increased the survival rate in mice after collagen-epinephrine-induced pulmonary thrombosis.

More advanced methods are based on the measurement of accumulation of radiolabeled platelet aggregates in the pulmonary circulation. In this approach, platelets are collected from a donor animal, labeled with a radioligand and injected into a recipient animal. Accumulation of platelets in the pulmonary circulation, resulting from injection of an agonist, is measured as an increased scintillation counts over the thoracic region of the animal. Tymvios et al. [36] studied dose-dependent responses to three platelet agonists (ADP, collagen and thrombin) in this model. To verify whether the responses were platelet-dependent, some of the animals were injected with aspirin. All tested agonists induced dose-dependent changes in platelet counts due to the accumulation of thrombi in the pulmonary vasculature, and aspirin was able to inhibit such collagen-induced responses. The location of platelet aggregates in the pulmonary bed was further confirmed histologically. This work confirmed that this model could be used to investigate the pharmacology of exogenous and endogenous modulators of platelet function.

Paul et al. [37] used this model to verify whether diabetes affects platelet reactivity in vivo and how this effect differs between animal models of type 1 and type 2 diabetes. Adenosine diphosphate (ADP) was used as a platelet agonist. Rats with streptozotocin (STZ)-induced diabetes served as a model of type 1 diabetes and Zucker diabetic fatty (ZDF) rats served as a model of type 2 diabetes. Platelets of the obese ZDF rats were significantly more responsive to ADP-induced aggregation in vivo than those of the lean ZDF rats. No such effect was observed in the STZ-treated rats. Interestingly, in vitro measurements of platelet reactivity revealed a different pattern of responses to ADP: no differences were observed between platelets of the ZDF obese and lean animals, while platelets from the STZ-treated rats were more reactive than those from the non-diabetic controls. This study shows that platelet reactivity measured under in vitro conditions may not reflect the actual functional status of platelets in the circulation.

In another work, which utilized this approach to assess the influence of functional state of the vascular wall on platelet reactivity, the authors assessed the contributions of cyclooxygenase-1 (COX-1) and cyclooxygenase-2 (COX-2) isoforms to platelet activation. The contribution of COX-2 activity to thrombosis gained special attention, since some of clinical trials have shown that drugs which selectively inhibit COX-2 (coxibs) augment the rate of thrombotic events. This effect was hypothesized to be due to the suppressing effect of coxibs on endothelial production of PGI_2_. To verify this hypothesis, Armstrong et al. [38] used a model of pulmonary thrombosis induced by collagen or the thromboxane A_2_ (TXA_2_) mimetic—U46619. The study was performed in two sets of conditions: upon the administration of a non-selective inhibitor of COX (diclofenac) or a selective COX-2 inhibitor (parecoxib). Parecoxib had no effect upon in vivo thrombus formation caused by either agonist. Hence, the study did not support the hypothesis that the inhibition of COX-2 in the vascular wall may acutely alter the local hemostatic environment in healthy patients.

The protocol which utilizes radiolabeled platelets in the pulmonary thromboembolism model provides reliable and reproducible outcomes in assessing platelet aggregation responses in vivo. Moreover, the responses are dose-dependent and graduated quantitative data could be obtained. This approach, however, has a drawback of artifactual platelet activation during the procedure of platelet isolation and labeling. However, according to Oyekan et al. [39] this activation only affects labeled platelets, which are a small portion of the recipient animal’s platelets, and therefore, the measurement of their accumulation reflects in fact the status of platelets of a recipient rather than the status of labeled platelets per se. Other disadvantages of this technique are the high prices demanded for radioisotopes and the disposal of radioactive animal remains.

An alternative approach, which is devoid of the above restrictions, was described by our group [40]. In this protocol, a decrease in systemic blood flow, resulting from an occlusion of pulmonary circulation, was monitored and recorded by a laser Doppler probe positioned over one of the peripheral blood vessels. This approach was validated by showing that blood flow cessation caused by ADP was less pronounced in platelet-depleted mice, and mice pretreated with either cangrelor or eptifibatide. Ex vivo imaging of selected organs from the mice with fluorescently labeled platelets revealed that ADP injection resulted in the significant accumulation of platelet occlusive aggregates only in the lungs. The method has been further used by our group in the research aimed at the potential application of polycationic polymers as drug carriers [41].

Systemic application of agonist is relatively less frequently used as a model of platelet activation than the methods based on an injury of the wall of blood vessel.

## 4. Studies on the Platelet Interaction with the Intact Vascular Wall

The involvement of platelets in the development of inflammation and atherosclerosis has been very extensively studied in recent decades [42]. The role of platelets in this process is to a large extent associated with their ability to interact with the vascular wall and to recruit other types of cells to it. When studying these processes, researchers are interested in assessing interactions of native platelets with the vascular wall, where the endothelium is mechanically intact but “activated.” Activation of the endothelium is a phenotype that is associated with decreased production of anti-adhesive mediators, such as nitric oxide or prostacyclin, and with increased expression of pro-adhesive receptors and ligands, such as ICAM, VCAM and vWF. Hence, protocols used in these studies do not include direct activation of platelets or disruption of the endothelial layer. Instead, the endothelium would be challenged with factors aimed at changing its phenotype. The extent of platelet interactions with endothelium is assessed by quantification of still images or movie sequences acquired by intravital microscopy. In order to visualize platelets, they are stained with fluorescent dyes. This can be performed either extracorporeally, when the platelets collected from donor animals are incubated with a fluorescent probe and infused to a recipient animal, or it can be achieved by the injection of platelet-specific antibodies to the experimental animal. Quantification of platelet interactions with the vascular wall is a challenging task. As will be shown in the examples presented below, the calculation of parameters of platelet motility strongly varies between various protocols.

Evaluation of platelet interactions with the vascular wall was used to test the hypothesis of the contribution of endothelial COX activity to platelet function. Buerkle et al. [43] studied the effects of selective COX-2 inhibitors on platelet adhesion to the vascular wall in hamsters pretreated with the selective COX-2 inhibitor NS-398. The animals were infused with calcein-labeled human platelets, and platelet interactions with arterioles in the dorsal skinfold chamber were recorded. Firm adhesion of a platelet to the vessel wall was defined when a platelet did not change its position for a period of at least 30 s. In case of other platelets, their velocities were calculated. It turned out that the selective COX-2 inhibition led to an increase in platelet interactions with the vascular wall. Therefore, the results suggest that it was an effect of a decreased production of endothelial prostacyclin. Interestingly, COX-2 inhibition in this model also increased the thrombotic vessel occlusion after the disruption of the vessel wall. These results are contradictory to the studies of Armstrong et al. [38], described above, which showed a lack of the effect of COX-2 inhibition on platelet aggregation in vivo in the model of pulmonary thromboembolism.

One of the aspects of endothelial dysfunction is a post-ischemic endothelial injury. Platelets were supposed to play an important role in this process. This notion was verified by Massberg et al. [44] in the in vivo model of ischemia/reperfusion. The experiments were performed in a model of ischemia of the small intestine in mice infused with fluorescently labeled platelets. After one-hour, ischemia interactions between platelets and the endothelium were monitored with the use of intravital fluorescence microscopy. Platelets were classified as free flowing, rolling (intermittent platelet adhesion) or adherent cells, according to their time of interaction. Rolling platelets were defined as platelets crossing the venular segment at a velocity significantly lower than the centerline velocity; their numbers were expressed as cells per second per vessel diameter. Adherent platelets were defined in each vessel segment as the number of objects that remained stationary for 30 s. Numbers of both rolling and adherent platelets were higher in the postischemic vessels. Platelet rolling and adhesion were impaired in the P-selectin-deficient mice, and in the mice injected with antibodies functionally blocking P-selectin, which pointed to an important role of this selectin in interactions of platelets with dysfunctional endothelium. When platelets from P-selectin-deficient mice were injected to wt mice, the platelet rolling and adhesion did not differ from that observed in wt mice, which showed that it is endothelial P-selectin which determines the process, while platelet P-selectin is not inevitable for these interactions.

The phenomenon of platelet adhesion to activated endothelium was explored in several other studies. In one of these works, Andre et al. [45] used a model of vessel stimulation with calcium ionophore A23187, which is commonly used as secretagogue of Weibel–Palade bodies, optionally with histamine. Mice were infused with fluorescently labeled platelets and the numbers of platelets rolling on or adhering to the vascular wall were measured in the mesenteric venules with the use of intravital microscopy. To verify the contributions of platelet receptors to this process, β_3_-deficient, vWF-deficient, P-selectin-deficient and GPIbα-deficient mice were used. Both calcium ionophore and histamine caused rapid platelet adhesion to the venule walls, and it was to a large extent diminished in vWF-deficient and GPIbα-deficient mice. P-selectin deficiency had only a minor effect and β_3_-deficiency did not affect the platelet adhesion. Therefore, in this model of endothelium activation, the interaction of platelet GPIbα with vWF secreted by the endothelium is the main mechanism responsible for platelet adhesion to the vascular wall.

Nowadays it is clear that the development of atherosclerosis is dependent, to a very high extent, on platelets. One of the first published studies which provided experimental evidence for this process was performed with the use of apolipoprotein E-deficient mice (ApoE^-/-^) that develop atherosclerotic lesions and are considered as the animal model of atherosclerosis [46]. Fluorescently labeled platelets were infused to the mice and the platelet adhesion in the carotid artery was evaluated with the use of intravital microscopy. Numbers of transient and firmly adhering platelets were assessed. Platelets were regarded as firmly adherent if they did not displace for at least 20 s and as transiently adherent if they were crossing the venular segment at a velocity significantly lower than the centerline vessel velocity. To assess how adhesion of platelets depends on the severity of lesions, mice in different age were studied. A significant increase in transient interactions of platelets with the vascular wall was observed in 8-week old ApoE^-/-^ mice when compared to wt mice. In 10-week old mice, this increase was significant also in the case of firmly adherent platelets. Importantly, such an increased platelet adhesion to the wall of the carotid artery preceded the occurrence of histological hallmarks of atherosclerotic alterations, suggesting that platelets take part in the early stages of atherosclerotic development. It is worth stressing that an increase in transient adhesions occurred before the occurrence of firm adhesions, which may suggest that such intermittent interactions of platelets with the endothelium are early hallmarks or even a cause of the transformation of the endothelial phenotype to its “activated” form. To determine which receptors contributed to the platelet adhesion, labeled platelets were preincubated with anti-GPIbα or anti-GPIIb-IIIa blocking antibodies prior to their infusion to mice. It was shown that GPIbα contributed to both transient and firm adhesion, whereas GPIIb-IIIa played an important role only in firm adhesion. Therefore, this study confirmed a crucial role of the GPIbα-vWF axis in platelet adhesion to the activated endothelium. To confirm that these interactions play a role in the development of atherosclerosis, ApoE^-/-^ mice were injected twice a week with anti-GPIbα for 12 weeks and the development of atherosclerotic lesions was evaluated. It was shown that the supplementation with antibodies significantly decreased the formation of atherosclerotic plaques. Altogether, this study provided evidence that platelets play an important role in the development of atherosclerosis in its early stages.

Another platelet receptor, whose contribution to the development of atherosclerosis has been described with the use of intravital microscopy, is F11R, also known as the junction adhesion molecule-A (JAM-A). It has been reported that the endothelial pool of this protein plays a crucial role in the development of atherosclerosis [47]. Babinska et al. [48] has shown that the long-term application of peptides which block homophilic interactions of this receptor to ApoE^-/-^ mice, decreased the formation of atherosclerotic plaques. In order to verify whether these peptides also block the platelet-endothelial interaction in this model, intravital microscopy of platelets was used. Platelets were labeled with the use of platelet-specific DyLight488-labeled anti-GPIbβ antibodies. Platelet tethering to the endothelium was analyzed by measuring the length of the track and time in which the distance was covered by the platelets. It was found that the peptides effectively decreased the interaction of platelets with the vascular wall in arterioles, but not in venules, suggesting that F11R/JAM-A-dependent interactions prevailed in high shear rates. Most of the interactions were transient and lasted shorter than one second. As was shown by Massberg [46], such short-lasting interactions dominate in the early stages of atherosclerosis.

Diabetes is considered as a state associated with endothelial dysfunction. However, there are only a few reports in the literature about interactions between platelets and the intact endothelium in animal models of diabetes. Therefore, our group investigated the pro-adhesive properties of the endothelium as an effect of streptozotocin (STZ)-induced diabetes and the roles of two main platelet receptors: GPIIb/IIIa and GPIb-IX-V in platelet-endothelial cell interactions [49]. In this study, platelets were labeled with the use of platelet specific anti-GPIbβ antibodies. Platelet adhesion to the vascular wall was assessed in STZ-diabetic animals with the use of in vivo microscopy. Two types of interactions between the platelets and endothelium were identified: one in which some of the objects were attached to the vascular wall and remained at the same point for a period of time, and another in which cells did not remain at one place and they rolled or slid. The results showed that platelets in a murine model of diabetes are more likely interacting with the intact vascular wall than those in healthy animals. By using specific antibodies blocking GPIbα, it was shown that these interactions were dependent on platelet GPIbα and the expression of vWF in endothelial cells, which was confirmed by previously published studies on the role of GPIbα-vWF axis in platelet interactions with the activated endothelium [50,51,52].

As long as the fact that platelets adhere to the activated endothelium is well established, the exact role of platelets in perpetuating inflammation is still not well understood. Although some in vitro studies exist that address these issues, there is still very little in vivo research which could help to understand this process. An interesting study that casts new light on this problem has been recently published by Zuchtriegel et al. [53]. The authors proposed the role of platelets in the mechanism by which leukocytes find their site of extravasation during inflammation. The inflammation was induced in the cremaster muscle of monocyto-reporter mice (CX3CR1GFP/+ mice) by C-C motif chemokine (CCL2), and interactions of platelets and leukocytes with endothelial cells were analyzed with the use of multichannel in vivo microscopy. It was shown that 30 min after CCL2 stimulation, platelets were the first adhering blood components at the inflamed endothelium, whereas neutrophils and inflammatory monocytes (iMOs) started to adhere one hour after the inflammation was elicited. Real-time confocal imaging allowed them to show intravascularly adherent platelets interacting with rolling neutrophils and iMOs by capturing them in the inflamed vascular wall. A crucial role of platelets in the process of extravasation of leukocytes was proven by showing that platelet depletion completely abolished the intravascular adherence and transmigration of neutrophils and iMOs. To understand the mechanisms underlying the interactions of platelets with endothelial cells, neutrophils and iMOs, animals received blocking antibodies or inhibitors directed against a series of platelet receptors and ligands. Blockade of vWF or GPIIbIIIa significantly reduced the number of adherent platelets to the inflamed endothelium, while the blockade of GPIbα, CD40L/CD154, CD40, P-selectin/CD62P, PSGL-1/CD162, PECAM-1/CD31 or ICAM-2/CD102 had no effect. The lack of the effect of GPIbα blockade on adhesion with a concomitant effect of vWF or GPIIbIIIa blockade suggests that it was the GPIIbIIIa-vWF axis rather than the GPIbα-vWF axis that was responsible for the interactions. The main finding of this study was that platelets play a role as pathfinders, which guide leukocytes into the site of extravasation in the microvasculature.

The increasing burden of evidence shows that platelets are important players in metastasis. When fluorescently stained cancer cells were injected to GFP mice, the formation of aggregates of platelets around these cells was observed in the liver in real-time with the use of intravital two-photon laser scanning microscopy [54]. In similar way, Labelle et al. [55] have shown that platelets form aggregates with tumor cells in lungs, and that platelet-derived TGFβ1 stimulates an invasive phenotype in tumor cells. The involvement of platelets in metastasis was assessed in the studies performed in lung allografts implanted in dorsal skin-fold chambers in mice [56]. Intravital microscopy revealed that in mice with depleted platelet count, the number of extravasated tumor cells in the lung microvasculature was lower than in the mice with a normal platelet count.

The experimental conditions of studies of platelets interactions with intact, activated vascular wall are very heterogenic. The main source of this variability is the way of induction of the pro-adhesive phenotype of endothelium which results from a diversity of pathological conditions which are being reproduced by these models. Additionally, the way of quantitation of platelet adhesion differs significantly between the protocols. Platelets interactions with vascular wall are not limited to a rolling but they tend to move in a stop-and-go fashion which may generate some discrepancies in terms of definition of adhered and non-adhered platelet. Therefore, comparison of the results of different studies is even more difficult than in experimental models of thrombosis.

## 5. Conclusions

Intravital methods allowed the researchers to better understand the numerous aspects of platelet biology, ranging from the mechanisms that govern platelet adhesion and aggregation during the thrombus formation to their contributions to the phenomena which are not directly associated with blood clotting, such as inflammation, atherosclerosis and metastasis. Although there are controversies regarding the relevance of some experimental models to the physiological conditions, it is undeniable that they provide better approximations of these conditions than in vitro approaches. However, there are several aspects of in vivo studies which still require development and improvement. One of them is the elaboration of biological models to better reflect pathophysiological conditions of thrombosis than the existing models. This also includes standardization and unification of the procedures between laboratories. Another aspect that needs to be addressed is the quantification of data obtained with the use of microscopic techniques. This applies specifically to the evaluation of platelet adhesion to the vascular wall. Standardization of criteria applied to calculations of immobilized and tethered platelets will facilitate comparison of the results between different laboratories.

Rapid development of microscopic imaging methods, and non-invasive macroscopic imaging techniques based on bioluminescence, magnetic resonance or computer tomography, will further improve in vivo studies. Therefore, it can be expected that intravital techniques will provide a tool for expanding our knowledge on platelet biology, and thus it will help to improve the development of novel therapies in cardiovascular diseases, inflammation or cancer.

At the same time, with great developments in sophisticated in vitro approaches such as lab-on-a-chip and organ-on-a-chip, it may be expected that a considerable part of research in the field of thrombosis will be performed in these setups rather than in animal models.
